# Supplementary material for: Chemical map-based prediction of nucleosome positioning using the Bioconductor package nuCpos
Source: BMC Bioinformatics. 2021 Jun 13;22:322. doi: 10.1186/s12859-021-04240-2 (PMC8201924; doi:10.1186/s12859-021-04240-2)

Chemical map–based prediction of nucleosome positioning using the Bioconductor package nuCpos

Supplemental Figures

Hiroaki Kato[1], Mitsuhiro Shimizu[2] and Takeshi Urano[1]

[1]Department of Biochemistry, Shimane University School of Medicine, Izumo, Shimane 693-8501, Japan

[2]Department of Chemistry, Graduate School of Science and Engineering, Program in Chemistry and Life Science, School of Science and Engineering, Meisei University, Hino, Tokyo 191-8506, Japan

Additional file 1: Figure S1

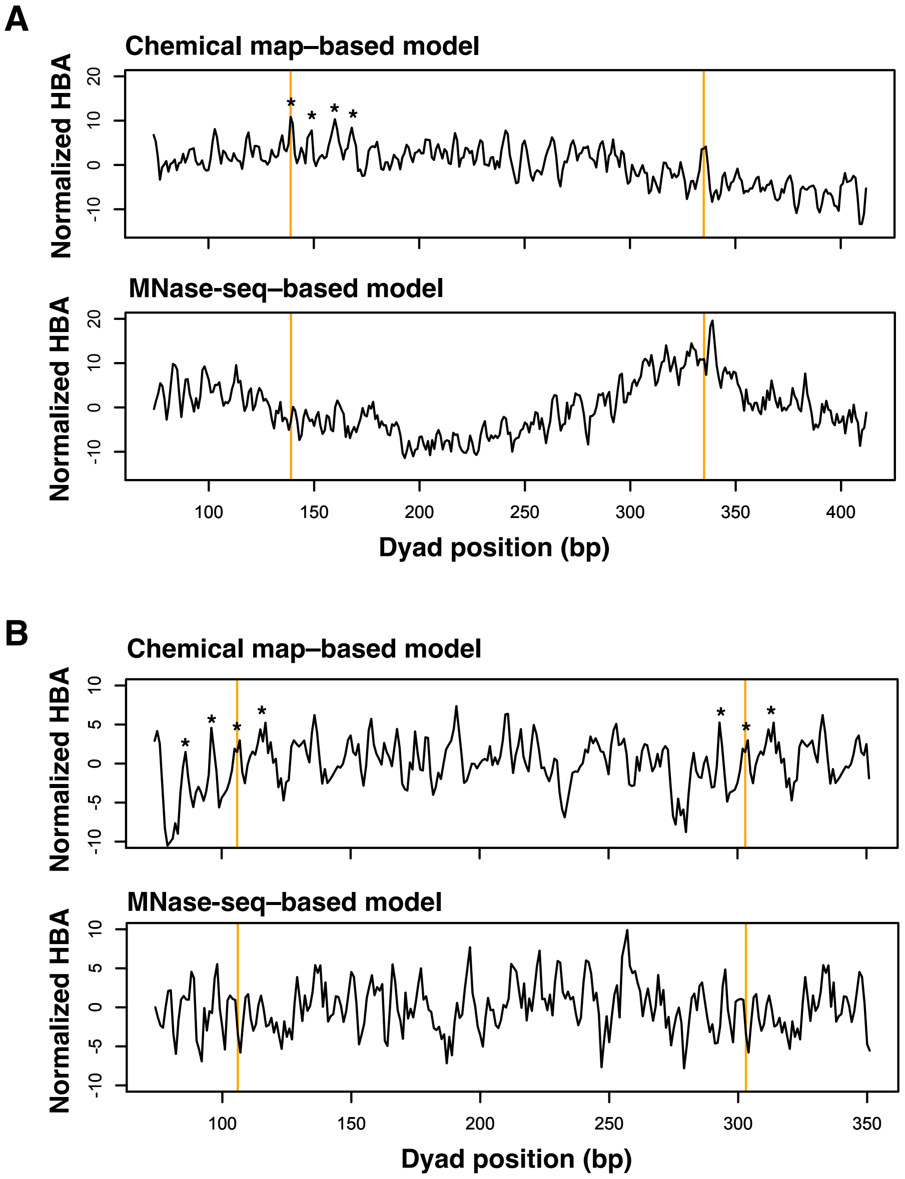

Additional file 1: Figure S2

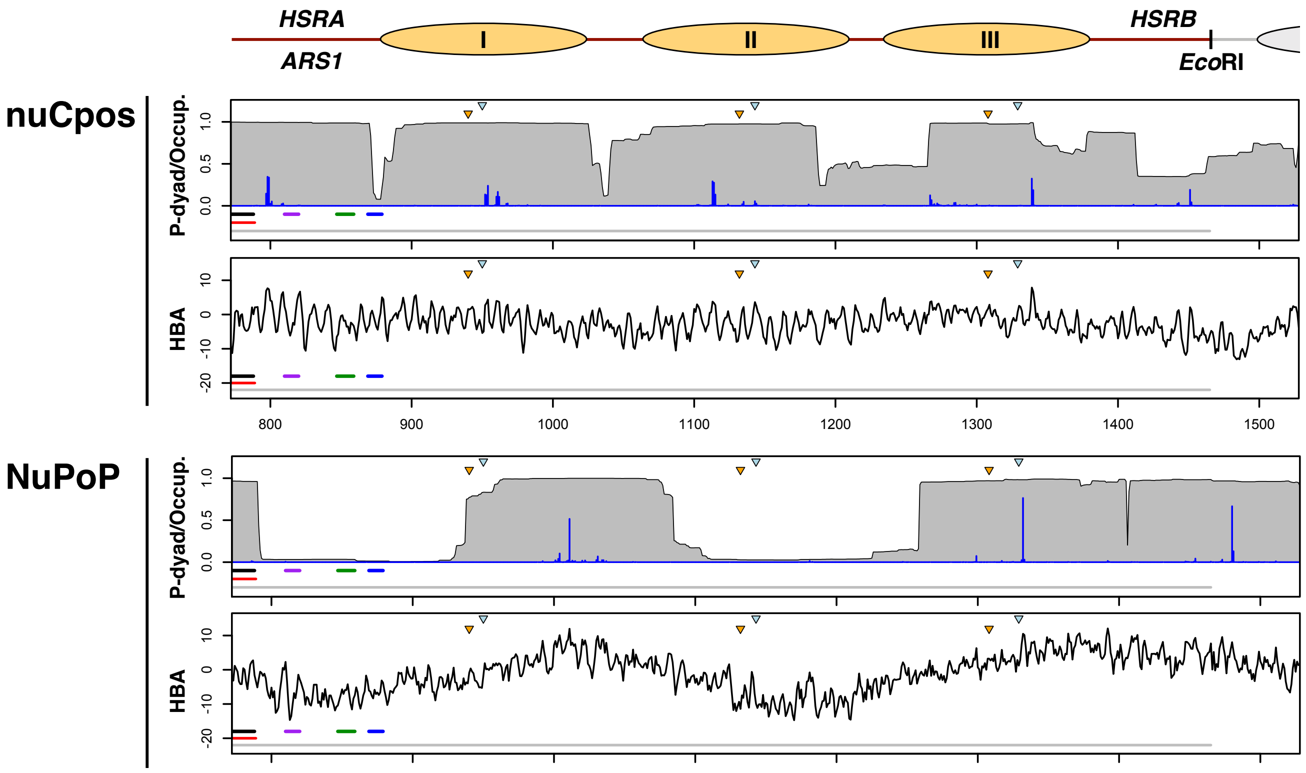

Additional file 1: Figure S3

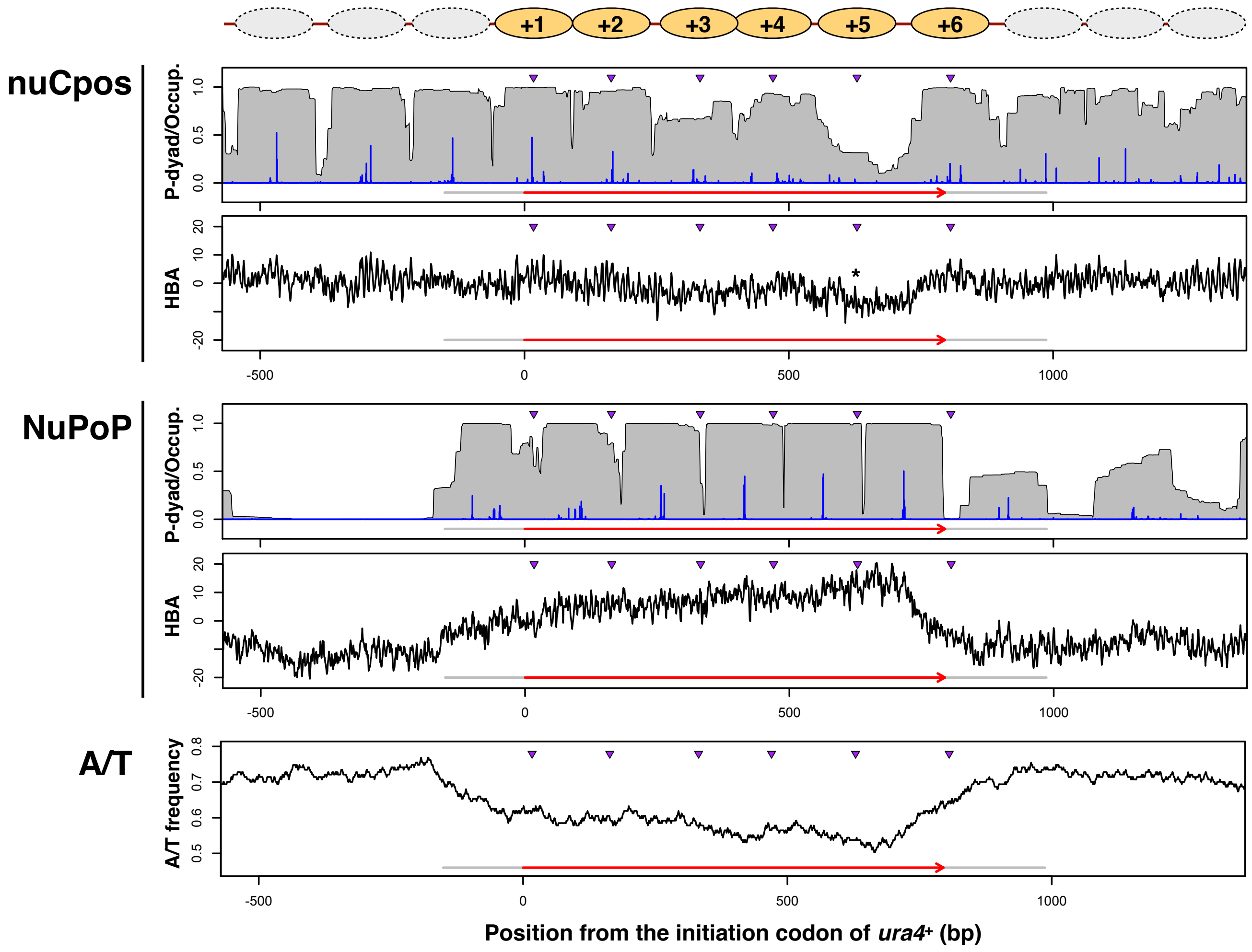

Additional file 1: Figure S4

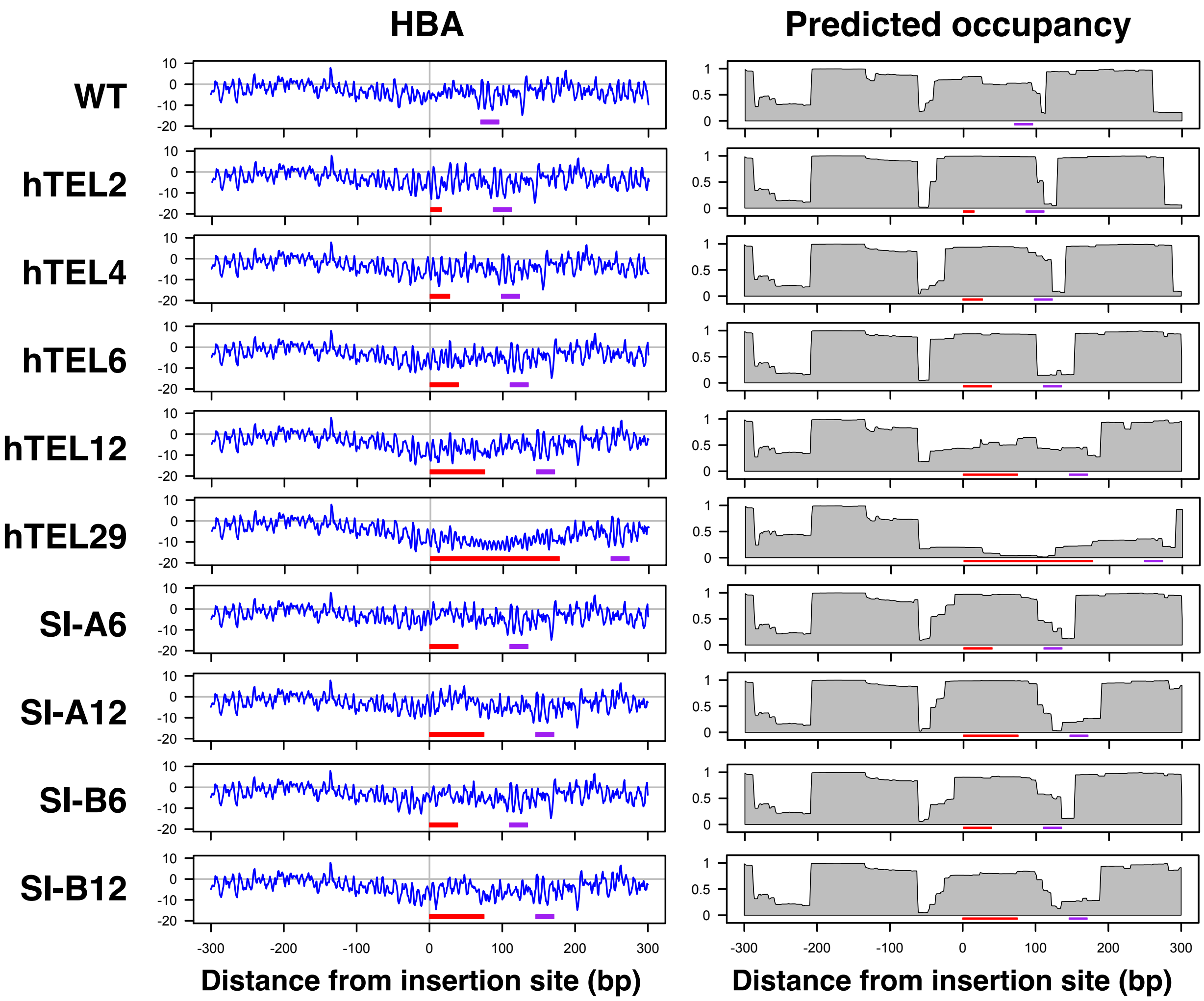

Additional file 1: Figure S5

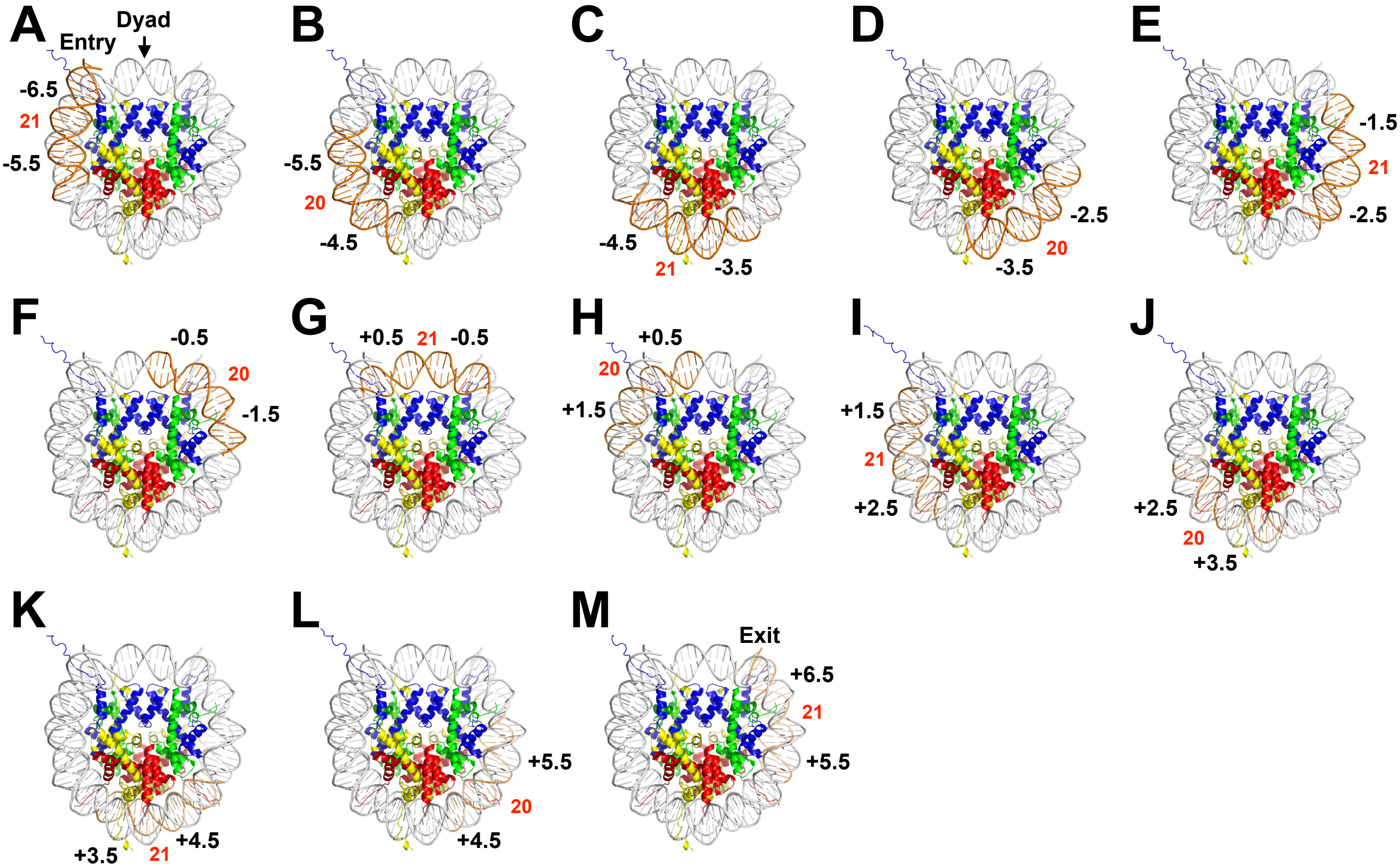

Supplement: Supplementary file 1 — Additional file 1. Supplemental figures. [file 12859_2021_4240_MOESM1_ESM.pdf]
