# Supplementary material for: Chemical map-based prediction of nucleosome positioning using the Bioconductor package nuCpos
Source: BMC Bioinformatics. 2021 Jun 13;22:322. doi: 10.1186/s12859-021-04240-2 (PMC8201924; doi:10.1186/s12859-021-04240-2)
Supplement: Supplementary file 2 — Additional file 2. Supplemental figure legeneds. [file 12859_2021_4240_MOESM2_ESM.pdf]

Chemical map–based prediction of nucleosome positioning using the Bioconductor package nuCpos

Hiroaki Kato[1], Mitsuhiro Shimizu[2] and Takeshi Urano[1]

[1]Department of Biochemistry, Shimane University School of Medicine, Izumo, Shimane 693-8501, Japan
[2]Department of Chemistry, Graduate School of Science and Engineering, Program in Chemistry and Life Science, School of Science and Engineering, Meisei University, Hino, Tokyo 191-8506, Japan

Supplementary Figure Legends

Additional file 1: Figure S1. HBA scores along *in vitro* nucleosome-forming sequences. HBA scores along nucleosome-forming sequences were calculated using chemical map–based and MNase-seq–based budding yeast models. Sequences analyzed were the 3'-LTR of MMTV (**A**) and the *Xenopus borealis* 5S rDNA dinucleosome-forming sequence (**B**). The scores were normalized by subtracting the mean value from the raw value on a per sequence basis. The HBA score for a given 147-bp nucleosome sequence was assigned to its dyad position. Orange vertical lines indicate the dyad positions of *in vitro* reconstituted nucleosomes: nucleotide positions 139 and 335 (**A**) and 106 and 303 (**B**). Asterisks indicate high-scoring HBA positions around the *in vitro* positioning sites. Note that for the 5S rDNA sequence, two identical sequences are joined at position 230, causing a difference in HBA scores around the left sides of the two *in vitro* nucleosome positions.

Additional file 1: Figure S2. Magnified view of prediction results for *TRP1ARS1*. Prediction results for the budding yeast *TRP1ARS1* mini-chromosome output by nuCpos and NuPoP (**Figure 3C**) were magnified, being centered at nucleosome II.

Additional file 1: Figure S3. Prediction results for the fission yeast *ura4*⁺ gene. Schematic representation of *in vivo* nucleosome positioning is shown above the plots. Nucleosomes numbered +1 to +6 are indicated as ovals. The top two panels show the prediction results output by nuCpos, whereas the next two panels show NuPoP results. The upper panel in each set shows predicted occupancy of nucleosomes (Occup., gray polygons) and probability that the tested 147-bp sequences are in the nucleosome state (P-dyad, blue vertical lines). Lower panels show HBA values for the tested 147-bp sequences calculated using the indicated models. The very bottom panel shows the A/T-frequency for the tested 147-bp sequences. Horizontal lines at the bottom of each plot indicate the 5'- and 3'-untranslated regions (colored in gray, nucleotide positions −151 to −1 and +795 to +986, respectively) and the protein-coding region (red, 0 to +794) of the *ura4*⁺ gene. Inverted triangles indicate the nucleosome centers determined by MNase-seq [1].

Additional file 1: Figure S4. Prediction of the effects of repeat insertion in the fourth nucleosome of the *TALS* minichromosome. Chemical map–based HBA scores and predicted nucleosomal occupancy were calculated for original and modified *TALS* sequences [2, 3]. The sequences were centered at the dyad position of the fourth nucleosome (0 bp), in which the indicated repeat sequences were inserted. Note: the position for the fourth nucleosome was predicted to shift to the right. Horizontal lines at the bottom of each plot indicate the *α2*-operator (purple) and the insert (red). hTEL stands for human telomeric repeat (5'-TTAGGG-3'); SI-A, sequence isomer-A (5'-TGTAGG-3'); SI-B, sequence isomer-B (5'-TGTGAG-3'). When telomeric DNA fragments are inserted at the center of the fourth nucleosome, shorter fragments (hTEL2 and hTEL4) were predicted to not affect nucleosome positioning. In contrast, longer fragments (hTEL12 and hTEL29) were predicted to severely compromise nucleosome formation. In addition, sequence isomers of the telomeric repeat (SI-A6, SI-A12, SI-B6, and SI-B12) were predicted to not inhibit nucleosome formation. These prediction results agreed with previous *in vivo* observations [2]. The hTEL6 insertion, which causes nucleosome depletion *in vivo*, appeared not

to affect nucleosome formation in the prediction, suggesting a limitation of the prediction.

Additional file 1: Figure S5. Thirteen overlapping nucleosomal DNA subsegments for local HBA calculation. Segments A through M are colored in orange on the nucleosome structure 1AOI [4]. The structures were drawn using MacPyMOL (v1.8.4.1). The base pair length (red) and corresponding superhelical locations (black) of each segment are indicated. The color of the right-half segments (H-M) is faded because they are behind the front helix.

References

1. Gonzalez S, Garcia A, Vazquez E, Serrano R, Sanchez M, Quintales L, Antequera F: **Nucleosomal signatures impose nucleosome positioning in coding and noncoding sequences in the genome.** *Genome Res* 2016, **26:**1532–1543.
2. Ichikawa Y, Morohashi N, Nishimura Y, Kurumizaka H, Shimizu M: **Telomeric repeats act as nucleosome-disfavouring sequences in vivo.** *Nucleic Acids Res* 2014, **42:**1541–1552.
3. Roth SY, Dean A, Simpson RT: **Yeast alpha 2 repressor positions nucleosomes in TRP1/ARS1 chromatin.** *Mol Cell Biol* 1990, **10:**2247–2260.
4. Luger K, Mader AW, Richmond RK, Sargent DF, Richmond TJ: **Crystal structure of the nucleosome core particle at 2.8 A resolution.** *Nature* 1997, **389:**251–260.
